# MKRN2-Mediated Degradation of IGF2BP3 Suppresses MYC and Enhances CDK4/6 Inhibitor Sensitivity in Bladder Cancer

**DOI:** 10.3390/cancers18132164

**Published:** 2026-07-06

**Authors:** Qi Pan, Qing Shi, Yubo Zhao, Tianxi Yu, Shiyu Bai, Haoran Zhu, Wei Zhang, Yaowei Li, Ziyi Liu, Haonan Li, Ziqi Wang, Zhichao Tong

**Affiliations:** 1 Department of Urology, Shanghai General Hospital, School of Medicine, Shanghai Jiao Tong University, Shanghai 200080, China; panqi0114@163.com; 2 NHC Key Laboratory of Molecular Probe and Targeted Theranostics, Harbin Medical University Cancer Hospital, Harbin Medical University, Harbin 150001, China; 3 Department of Urology, Harbin Medical University Cancer Hospital, Harbin 150001, China; 4 Department of Urology, Second Affiliate Hospital of Harbin Medical University, Harbin 150001, China; 5 Department of Pharmacology, College of Pharmacy, Harbin Medical University, Harbin 150001, China; 6 Department of Cystoscope Center, Harbin Medical University Cancer Hospital, Harbin 150001, China; 7 Heilongjiang Provincial Key Laboratory of Basic Medical Sciences in Urology Cancer, Harbin Medical University Cancer Hospital, Harbin 150001, China; 8 Biobank, Harbin Medical University Cancer Hospital, Harbin 150001, China; 9 Department of Urogenital Medical Oncology, Harbin Medical University Cancer Hospital, Harbin 150001, China

**Keywords:** bladder cancer, CDK4/6 inhibition, proliferation, IGF2BP3, MYC, MKRN2

## Abstract

Background: CDK4/6 inhibitors induce G1/S cell-cycle arrest in bladder cancer; however, adaptive resistance limits their therapeutic efficacy. The role of the m^6^A reader IGF2BP3 in regulating sensitivity to CDK4/6 inhibition remains largely unknown. Methods: Transcriptomic profiling was performed in palbociclib-treated bladder cancer cell lines (T24, RT112, and UMUC-3) to identify m^6^A regulators associated with drug response. The expression and clinical significance of *IGF2BP3* were evaluated using The Cancer Genome Atlas (TCGA) data and an independent clinical cohort. Gain- and loss-of-function assays were conducted to investigate the effects of IGF2BP3 on cell proliferation and cell-cycle progression. Mechanistic studies, including RNA-binding, mRNA stability, ubiquitination, and in vivo tumorigenesis assays, were performed to elucidate the underlying regulatory network. Results: IGF2BP3 was identified as the only m^6^A regulator differentially expressed following palbociclib treatment. IGF2BP3 expression was significantly elevated in bladder cancer tissues compared with normal tissues and was associated with poor prognosis and Ki67 positivity. Functionally, IGF2BP3 overexpression (OE) promoted G1/S transition, increased MYC and downstream cell-cycle regulators, and partially rescued palbociclib-induced cell-cycle arrest, whereas IGF2BP3 knockdown (KD) suppressed cell proliferation in an MYC-dependent manner. Mechanistically, IGF2BP3 bound to MYC mRNA in an m^6^A-dependent manner and enhanced its stability. Furthermore, MKRN2 was identified as an E3 ubiquitin ligase that directly interacted with IGF2BP3, promoted its ubiquitination, and facilitated its proteasomal degradation. In vivo, MKRN2 co-overexpression attenuated IGF2BP3-driven tumor growth and synergized with palbociclib to maximally suppress tumor volume, reduce MYC and Ki67 expression, and induce apoptosis. Conclusions: These findings establish the MKRN2–IGF2BP3–MYC axis as a critical regulator of CDK4/6 inhibitor sensitivity in bladder cancer. Targeting IGF2BP3 or enhancing MKRN2 activity may represent a promising strategy to overcome adaptive resistance and improve the therapeutic efficacy of CDK4/6 inhibitors.

## Simple Summary

This study identifies IGF2BP3 as the sole m^6^A reader dynamically regulated upon CDK4/6 inhibition in bladder cancer, where its high expression correlates with poor prognosis and increased proliferation. Mechanistically, IGF2BP3 stabilizes m^6^A-modified MYC transcripts to sustain G1/S progression and attenuate palbociclib-induced cytostasis, establishing a functional IGF2BP3–MYC axis that drives drug tolerance. The E3 ubiquitin ligase MKRN2 directly binds, ubiquitinates, and promotes proteasomal degradation of IGF2BP3, thereby limiting MYC expression and cell-cycle progression. MKRN2 overexpression synergizes with palbociclib to suppress tumor growth, reduce MYC/Ki67 expression, and induce apoptosis in vivo, effectively overcoming IGF2BP3-mediated resistance. Collectively, the MKRN2–IGF2BP3–MYC axis represents a novel post-translational mechanism modulating CDK4/6 inhibitor sensitivity, providing a rationale for biomarker-guided stratification and combination therapies targeting this pathway in bladder cancer.

## 1. Introduction

Bladder cancer displays persistent proliferative drive and remains therapeutically difficult to control, particularly once tumors acquire tolerance to cytostatic interventions [1]. Although chemotherapy, targeted therapy, and immune checkpoint inhibitor (ICI)-based immunotherapy, particularly PD-1/PD-L1 inhibitors, have improved the treatment of bladder cancer, therapeutic resistance remains a major clinical challenge [2,3], emphasizing the need to define actionable determinants of treatment response [4]. A central challenge is that bladder tumors can retain core cell-cycle circuitry while exhibiting markedly variable pharmacologic dependency on it, resulting in heterogeneous sensitivity to pathway-directed therapies [5].

Cyclin-dependent kinase 4/6 (CDK4/6) inhibitors, such as palbociclib, restrict G1/S progression by limiting RB phosphorylation and suppressing E2F-driven transcription [6]. Preclinical studies support activity in bladder cancer [7], but variable response patterns and rapid adaptation suggest that upstream or parallel regulatory layers can maintain cell-cycle transcription even when CDK4/6 is inhibited [8,9,10,11]. Defining these buffering mechanisms is essential for two reasons: they can explain de novo insensitivity [12], and they can reveal rational combinations that convert transient cytostasis into durable control [13,14].

The N^6^-methyladenosine (m^6^A) RNA modification machinery represents an attractive candidate buffering layer because it can reshape oncogenic states by controlling RNA fate [15] and, potentially, by intersecting with protein-level regulation [16]. IGF2BP proteins are prominent m^6^A readers with established roles in malignant progression [17] and drug response across tumor types [18]. However, whether IGF2BP3 actively rewires CDK4/6 inhibitor response in bladder cancer, rather than passively reflecting a proliferative state, remains unclear. Notably, MYC is a plausible convergence point: MYC orchestrates cell-cycle progression and transcriptional amplification [19], and its abundance is tightly governed by multiple mechanisms. Intriguingly, the stability of IGF2BP3 itself is subject to ubiquitin–proteasome regulation, and recent evidence identifies the E3 ubiquitin ligase MKRN2 as a direct mediator of IGF2BP3 degradation [20,21]. Given that IGF2BP3 is known to stabilize MYC transcripts in an m^6^A-dependent manner, we hypothesize that MKRN2-mediated turnover of IGF2BP3 may serve as an upstream checkpoint that governs MYC abundance and, consequently, modulates the sensitivity of bladder cancer cells to CDK4/6 inhibition.

Here, we identify IGF2BP3 as a determinant of attenuated response to CDK4/6 inhibition and define a mechanistic link between an m^6^A reader and MYC proteostasis via MKRN2-mediated degradation. By transcriptome profiling across three bladder cancer cell lines treated with palbociclib, we nominated *IGF2BP3* as the sole m^6^A regulator emerging from a shared differential expression signature. We further demonstrate that IGF2BP3 is clinically enriched and associated with adverse outcomes, and that IGF2BP3 abundance closely tracks proliferative activity in tumors. Mechanistically, MKRN2 directly binds to and promotes ubiquitination and proteasomal degradation of IGF2BP3, thereby limiting IGF2BP3 protein abundance. When MKRN2 is downregulated or IGF2BP3 is overexpressed, accumulated IGF2BP3 enhances *MYC* mRNA stability, leading to increased MYC protein expression, sustained downstream cell-cycle transcription, and reduced sensitivity to CDK4/6 inhibition. Genetic epistasis in vivo places MKRN2 as a critical upstream node of this circuit. Together, these findings establish an MKRN2–IGF2BP3–MYC axis that buffers CDK4/6 inhibition and provide a framework for biomarker-guided stratification and rational combinations in bladder cancer.

## 2. Materials and Methods

### 2.1. Online Databases and Associated Analyses

The Cancer Genome Atlas (TCGA-BLCA, https://cancergenome.nih.gov/ accessed on 25 August 2025) database was employed to analyze the expression profile of *IGF2BP3* and the expression correlation between *IGF2BP3* and *MYC* in bladder cancer (BC) tissues.

### 2.2. Clinical Data Analysis

This study included 20 patients with primary bladder cancer who underwent radical cystectomy at Harbin Medical University Cancer Hospital between January 2024 and January 2025. Written informed consent was obtained, and the protocol was approved by the hospital’s Ethics Committee (KY2024-100), complying with the Declaration of Helsinki. Clinical characteristics are summarized in Supplementary Table S1.

### 2.3. Cell Lines and Cultures

SV-HUC-1, seven bladder cancer cell lines (T24, UMUC-3, 5637, HT-1197, 647-V, J82, EJ), and HEK-293T were sourced from the National Collection of Authenticated Cell Cultures. UMUC-3, 647-V, J82, and HEK-293T were cultured in DMEM; the rest in RPMI-1640. All media contained 10% fetal bovine serum (FBS) and 1% penicillin/streptomycin. Three lines were treated with 1 μM palbociclib for 48 h.

### 2.4. Cell Proliferation and Viability Assays

Cell proliferation was assessed using the CCK-8 assay (GK10001, Glpbio, Montclair, CA, USA). Cells were seeded at a density of 3000 cells per well in 96-well plates and continuously cultured for 24, 48 and 72 h after treatment. Subsequently, CCK-8 reagent was added to each well, followed by a 2 h incubation at 37 °C, and cell viability was determined by measuring absorbance values.

### 2.5. 5-Ethynyl-2′-Deoxyuridine (EdU) Assays

EdU incorporation, a widely used assay for evaluating cell proliferation, was performed using an EdU Detection Kit (C0071S, Beyotime Biotechnology, Shanghai, China) with minor modifications to the manufacturer’s protocol. Fluorescent images were acquired using a fluorescence microscope, and the ratio of EdU-positive cells was quantified using ImageJ v1.54f software.

### 2.6. Clone Formation Assay

Cells were seeded in 6-well plates at 50 cells/well and treated the next day. After 2 weeks, cells were washed twice with phosphate-buffered saline (PBS), fixed in 4% paraformaldehyde for 15 min, stained with 0.1% crystal violet for 30 min, rinsed with double-distilled water, and air-dried.

### 2.7. Cell-Cycle Assays

Cells were digested by trypsin and cell pellets were obtained by centrifugation and then resuspended in ice-cold PBS. Then ice-cold 90% ethanol was added dropwise to the centrifuge tube under constant vortexing to prevent cells from clumping. Cells were centrifuged for 5 min at 2000 rpm and 4 °C after washing twice with ice-cold PBS and adding 200–400 μL 7-ADD solution (final concentration, 25 mg/mL) to each centrifuge tube. Finally, the samples were assayed on a flow cytometer (Beckman, Brea, CA, USA).

### 2.8. Terminal Deoxynucleotidyl Transferase dUTP Nick End Labeling (TUNEL) Assay

The TUNEL assay was performed on mouse xenograft sections using a Beyotime kit (C1089, Shanghai, China). After deparaffinization, rehydration and proteinase K digestion, sections were incubated with labeling mix at 37 °C in the dark. Sections were then incubated with streptavidin–TRITC or horseradish peroxidase (HRP) chromogen, counterstained with DAPI, mounted, and imaged. TUNEL-positive cells were quantified to assess apoptosis.

### 2.9. Lentivirus Construction and Infection

Expression plasmids were constructed by cloning wild-type or mutant cDNAs into pcDNA3.1(+) or pCDH-CMV-MCS-EF1-Puro. Lipofectamine transfection was used for transient assays. For stable overexpression/knockdown, pCDH or pLKO.1-puro shRNA vectors were generated. Lentivirus was produced in HEK293T cells with psPAX2 and pMD2.G, then used to infect RT112 and UMUC-3 cells. Stable lines were selected with puromycin. Constructs were from Addgene (Watertown, MA, USA) or custom-made, and verified by sequencing.

### 2.10. Western Blot (WB)

Cells at 70–80% confluence were lysed in radioimmunoprecipitation assay (RIPA) buffer containing PMSF and protease inhibitors. Protein concentration was measured with a BCA kit (Beyotime, P0012). Samples were normalized, denatured at 100 °C for 10 min, separated using an EpiZyme PAGE kit (PG110), and transferred to polyvinylidene fluoride (PVDF) membranes. Membranes were blocked with 5% non-fat milk for 2 h, then incubated overnight at 4 °C with primary antibodies: IGF2BP3 (proteintech, 14642-1-AP, 1:2000), MKRN2 (proteintech, 12238-1-AP, 1:2000), MYC (proteintech, 10828-1-AP, 1:2000), Skp2 (abcam, ab183039, 1:2000), Cyclin A (proteintech, 18202-1-AP, 1:2000), Cyclin E (proteintech, 11554-1-AP, 1:1000), E2F1 (abcam, ab288369, 1:1000), RB1 (proteintech, 10048-2-Ig, 1:2000), ph-RB1 (abcam, ab184796, 1:1000), ubiquitin (abcam, ab134953, 1:2000), and GAPDH (proteintech, 60004-1-Ig, 1:5000). After washing, blots were incubated with secondary antibodies for 1 h, and signals were detected using an Li-Cor Odyssey system (LI-COR Biosciences, Lincoln, NE, USA).

### 2.11. Ubiquitination Assay

For exogenous ubiquitination, Ni-NTA pull-down was performed in HEK293T cells co-transfected with Flag-HA-MKRN2, Myc-IGF2BP3 and His-Ub. After MG-132 treatment, cells were lysed in denaturing buffer with inhibitors, and ubiquitinated proteins were enriched by Ni-NTA beads. For endogenous ubiquitination, RT112 and UMUC-3 cells with stable MKRN2 knockdown were treated with MG-132, lysed in RIPA buffer with inhibitors, and immunoprecipitated with anti-IGF2BP3 antibody. Ubiquitination in both groups was detected by Western blot.

### 2.12. Cycloheximide (CHX) Chase Assay for Protein Stability

IGF2BP3 protein stability was measured via CHX chase assay. RT112 and UMUC-3 cells were grouped as shCtrl and MKRN2 KD. At log phase, cells were treated with 100 μg/mL CHX and harvested at 0, 1, 2, and 4 h. IGF2BP3, MKRN2, and GAPDH levels were detected by Western blot. IGF2BP3 levels were normalized to the 0 h time point (set as 100%), and degradation curves were generated.

### 2.13. Co-Immunoprecipitation (Co-IP)

Protein concentrations were determined first. Lysates were incubated with primary antibodies overnight at 4 °C to form immune complexes. A portion was boiled with 5× loading buffer (Beyotime, P0015) as the input. Magnetic beads were added to the remaining lysate, rotated for 20 min at room temperature, and centrifuged at 1400 rpm for 1 min at 4 °C. Beads were washed five times with lysis buffer, then boiled with loading buffer for Co-IP and IP samples. All samples were analyzed by Western blot.

### 2.14. RNA Immunoprecipitation (RIP)

RIP assays were conducted using a Beyotime kit (RN1001). RT112 and UMUC-3 cells were seeded in 10 cm dishes, harvested 48 h post-transfection, and resuspended in nuclease-free PBS. Lysates were incubated with Protein A/G beads conjugated to anti-IGF2BP3 or IgG control at 4 °C for 3 h. Bead–RNP complexes were washed three times, and bound RNA was extracted for quantitative real-time polymerase chain reaction (qRT-PCR).

### 2.15. m^6^A-RNA Immunoprecipitation (Me-RIP)

Me-RIP was performed using an m^6^A antibody (CST, #56593) to pull down m^6^A-modified *MYC* mRNA. Total RNA from RT112 and UMUC-3 cells was purified with an NEB polyA Spin kit. Purified RNA was incubated overnight at 4 °C with Protein A/G beads bound to m^6^A or IgG antibody, in the presence of protease and RNase inhibitors. Bound RNA was extracted and analyzed by qRT-PCR. For m^6^A-related proteins, bead–RNP complexes were separated by sodium dodecyl sulfate–polyacrylamide gel electrophoresis (SDS-PAGE) and analyzed by Western blot.

### 2.16. RNA Extraction

Total RNA was extracted following the standard protocols of the Tiangen (DP419) RNA Extraction Kit. RNA concentration was measured using a NanoDrop (ThermoFisher, Waltham, MA, USA) spectrophotometer. Based on the concentration determination results, one portion of the RNA was submitted for transcriptome sequencing; the other portion was reverse-transcribed into complementary DNA (cDNA) using the Yeasen Reverse Transcription Kit (Shanghai, China) and stored at −80 °C for subsequent experiments.

### 2.17. qRT-PCR Assay

Total RNA was extracted using an RNA extraction kit (BIQFLUX, Durham, NC, USA) and reverse-transcribed using the Reverse Transcription kit (11150ES60, YESEN, Shanghai, China). Real-time PCR was performed using the SYBR Green Realtime PCR Master Mix (QPK-201T, Toyobo, Osaka, Japan) following the manufacturer’s instructions. PCR conditions were as follows: 95 °C for 5 s, 55 °C for 10 s, and 72 °C for 15 s, for 40 cycles. The primer sequences were as follows (5′→3′):

IGF2BP3 forward primer: TATATCGGAAACCTCAGCGAGA.

IGF2BP3 reverse primer: GGACCGAGTGCTCAACTTCT.

MYC forward primer: GGCTCCTGGCAAAAGGTCA.

MYC reverse primer: CTGCGTAGTTGTGCTGATGT.

MKRN2 forward primer: AGGAAGTCAGTGCCTATTCTCA.

MKRN2 reverse primer: TGGTCATATCTGCACCGAGTT.

GAPDH forward primer: GGAGCGAGATCCCTCCAAAAT.

GAPDH reverse primer: GGCTGTTGTCATACTTCTCATGG.

### 2.18. RNA Stability Assay

To assess mRNA stability, cells were treated with 1 µg/mL actinomycin D (ActD). RNA was then isolated at regular intervals and subjected to quantitative reverse transcription-polymerase chain reaction (qRT-PCR). The half-life was determined by plotting the decay curve of mRNA after ActD treatment. This method allows for accurate evaluation of mRNA stability.

### 2.19. Chromatin Immunoprecipitation Assay (ChIP)

ChIP assays were performed using a Beyotime kit (P2078) with 2 × 10^7^ cells. Cross-linked chromatin was sonicated into 200–500 bp fragments, then immunoprecipitated with anti-MYC or normal IgG (Proteintech, Wuhan, China). Purified DNA (28104, Qiagen, Hilden, Germany) was analyzed by qRT-PCR (Toyobo, QPK-201T). Assays were repeated ≥3 times.

### 2.20. Immunohistochemical Staining (IHC)

IHC was performed on human bladder cancer and mouse xenograft paraffin sections (4 μm). Sections were dewaxed, dehydrated, and antigen-retrieved. After blocking for 2 h, primary antibodies (IGF2BP3 (proteintech, 14642-1-AP, 1:200), MKRN2 (proteintech, 12238-1-AP, 1:200), MYC (proteintech, 10828-1-AP, 1:200), Ki67 (proteintech, 27309-1-AP, 1:200)) were applied overnight at 4 °C. Secondary antibodies (Abcam, Shanghai, China, ab6721, ab205719, 1:200) were incubated for 1 h at room temperature, followed by DAB development, hematoxylin counterstaining, dehydration, clearing, and mounting. Immunohistochemical staining was evaluated independently by two experienced pathologists who were blinded to the clinical information. Three representative high-power fields (×400) were randomly selected from each section after excluding areas with necrosis, hemorrhage, and edge artifacts. At least 500 tumor cells were evaluated per specimen. Staining intensity was scored as 0 (negative), 1 (weak), 2 (moderate), or 3 (strong). The percentages of tumor cells showing weak, moderate, and strong staining were recorded separately. The H-score was calculated using the formula H-score = (1 × % of weakly stained cells(+)) + (2 × % of moderately stained cells(++)) + (3 × % of strongly stained cells(+++)), yielding a final score ranging from 0 to 300. Discrepant cases were reviewed jointly until a consensus was reached.

### 2.21. Xenograft Tumor Model

Male BALB/c nude mice (6 weeks old) were used for the xenograft experiments. Stable RT112 cell lines (control, IGF2BP3 overexpression, and IGF2BP3/MKRN2 co-overexpression) were established. Log-phase cells (5 × 10^6^ cells in 100 μL PBS) were subcutaneously injected into the right flank of each mouse. When tumors became palpable (day 7 after implantation), mice were randomly assigned to six groups (*n* = 5 per group): control, IGF2BP3-OE, palbociclib, IGF2BP3-OE + palbociclib, IGF2BP3/MKRN2-OE, and IGF2BP3/MKRN2-OE + palbociclib. Palbociclib (MedChemExpress, Shanghai, China, HY-50767, 75 mg/kg) was administered once daily by oral gavage. Body weight and tumor volume (length × width^2^/2) were measured weekly throughout the experiment. All mice were euthanized on day 28, and tumors were harvested, photographed, weighed, and fixed in formalin for immunohistochemical analysis.

### 2.22. Statistical Analysis

Statistical analyses were performed using R software (Version 4.0.0) or GraphPad Prism software (Version 9.0.0). Comparisons between two groups were conducted using the two-tailed Student’s *t*-test. Categorical data were evaluated by the chi-square test. Survival curves were plotted using the Kaplan–Meier method, and survival data were analyzed using univariate and multivariate Cox regression analyses. A *p* value < 0.05 was considered statistically significant.

## 3. Results

### 3.1. A Palbociclib-Perturbation Screen Nominates IGF2BP3 as an m^6^A-Linked Determinant of Attenuated CDK4/6 Inhibitor Response and Poor Patient Outcome

**Figure 1:**
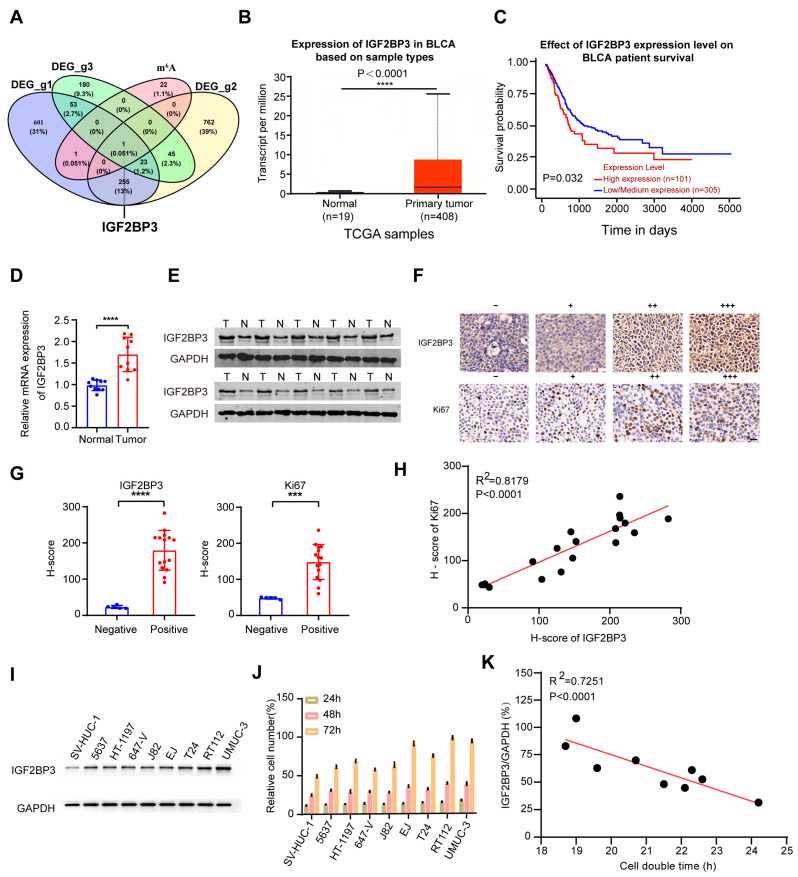
A palbociclib-perturbation screen nominates *IGF2BP3* as an m^6^A-linked determinant of attenuated CDK4/6 inhibitor response and poor patient outcome. (**A**) Venn diagram of DEGs and m^6^A regulators, with *IGF2BP3* as the sole overlapping gene. (**B**) TCGA: *IGF2BP3* mRNA higher in bladder cancer (T = 408) vs. normal (*n* = 19). (**C**) High *IGF2BP3* correlates with shorter OS (log-rank). (**D**,**E**) qRT-PCR and Western blot confirm higher *IGF2BP3* in clinical tumors vs. Normal, see Supplementary WB gels for details. (**F**) IHC: IGF2BP3 absent in normal tissues, variable expression in tumor cytoplasm/nucleus; Ki67 as proliferation marker (scale bar = 20 μm). (**G**,**H**) IGF2BP3 H-score positively correlates with Ki67 positivity, the red lines in panels represent linear regression fit lines to show the linear correlation between the IGF2BP3 and Ki67 in scatter plot. (R^2^ = 0.8179, *p* < 0.0001). (**I**) Western blot: IGF2BP3 absent in SV-HUC-1, variable in 8 bladder cancer lines. (**J**,**K**) IGF2BP3 expression inversely correlates with doubling time, the red lines in panels represent linear regression fit lines to show the linear correlation between the IGF2BP3/GAPDH and cell double time in scatter plot (R^2^ = 0.7251, *p* < 0.0001). The original WB membranes are provided in Supplementary WB gels. *n* = 3; mean ± SD; *** *p* < 0.001, **** *p* < 0.0001.

To identify m^6^A-related regulators associated with variable response to CDK4/6 inhibition in bladder cancer, we performed transcriptome profiling in three bladder cancer cell lines (T24, RT112 and UMUC-3) treated with palbociclib (1 μM) for 48 h, with untreated cells as controls [9] (Supplementary Figure S1A). Global expression distributions were highly concordant across the 18 transcriptomic profiles, with the majority of genes expressed at low-to-moderate levels and a smaller subset exhibiting high expression, consistent with high-quality sequencing (Supplementary Figure S1B). Differential expression analysis of control groups and drug-exposed groups of the three cell lines revealed that a total of 24 genes exhibited significant alterations. Meanwhile, we presented the top 25 upregulated and downregulated differentially expressed genes (DEGs) across the three sets of sequencing data (|log_2_FC| ≥ 2, *p* < 0.05; Supplementary Figure S1C), forming a compact palbociclib-response signature. Notably, intersecting this signature with a curated panel of 24 canonical m^6^A regulators yielded a single overlapping candidate, *IGF2BP3* (Figure 1A; Supplementary Table S2), nominating *IGF2BP3* as a putative m^6^A-linked determinant associated with attenuated response to CDK4/6 inhibition.

We next evaluated the clinical relevance of *IGF2BP3*. In TCGA bladder cancer data, *IGF2BP3* mRNA was significantly elevated in tumor tissues compared with adjacent normal tissues (Figure 1B), consistent with recent multi-omics findings in muscle-invasive bladder cancer [22]. Kaplan–Meier analyses further demonstrated that high IGF2BP3 expression was associated with shorter overall survival (OS) (Figure 1C). In an independent cohort of 20 clinically collected bladder cancer specimens, IGF2BP3 abundance was increased in tumor relative to normal controls at both transcriptome and protein levels (Figure 1D,E). Immunohistochemistry revealed minimal IGF2BP3 staining in adjacent normal tissues but variable cytoplasmic and nuclear staining in tumor cells (Figure 1F). Importantly, IGF2BP3 levels tightly tracked tumor proliferative activity: IGF2BP3 staining intensity strongly correlated with Ki67 positivity (R^2^ = 0.8179, *p* < 0.0001; Figure 1G,H). Extending these observations to cell models, IGF2BP3 was undetectable in non-malignant SV-HUC-1 cells but expressed at varying levels across eight urothelial carcinoma cell lines (Figure 1I). Across these eight lines, higher IGF2BP3 expression correlated with shorter population doubling time (Figure 1J,K). Collectively, these data position IGF2BP3 as a clinically enriched proliferation-associated factor and nominate it from an unbiased palbociclib-perturbation screen as a candidate regulator of CDK4/6 inhibitor response in bladder cancer.

### 3.2. IGF2BP3 Sustains Cell-Cycle Drive and Partially Buffers Palbociclib-Induced G1 Arrest

**Figure 2:**
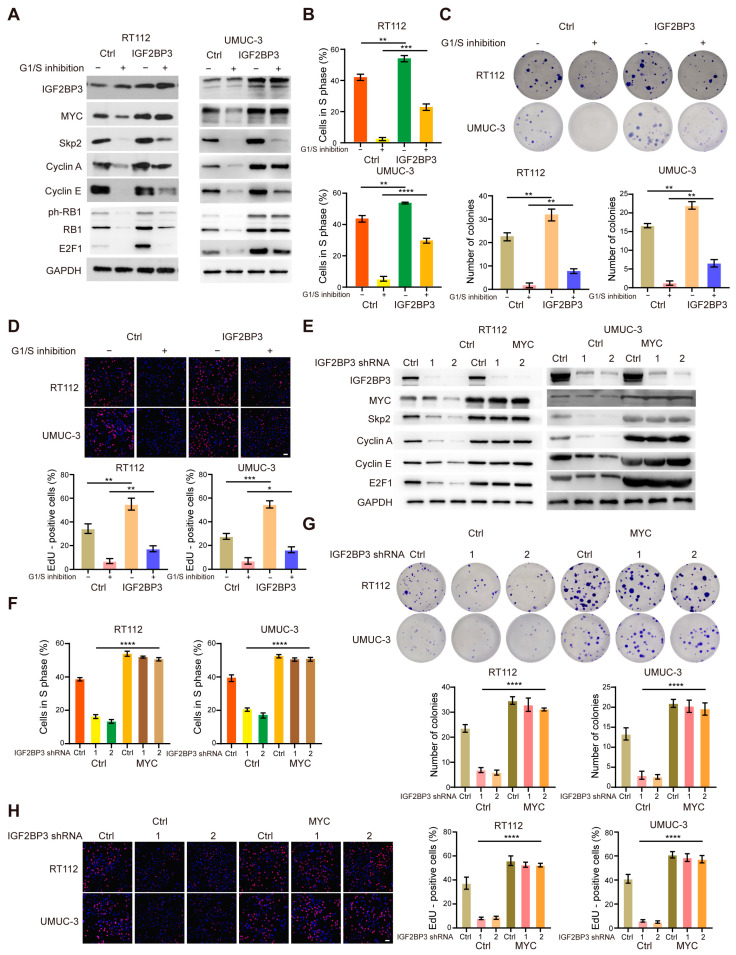
IGF2BP3 sustains cell-cycle drive and partially buffers palbociclib-induced G1 arrest. (**A**) Western blot: IGF2BP3-OE upregulates MYC and cell-cycle genes; palbociclib (PD-0332991) suppresses these in controls, an action partially reversed by IGF2BP3-OE. (**B**) Cell cycle: IGF2BP3-OE promotes progression and rescues palbociclib-induced G1 arrest. (**C**,**D**) Colony formation and EdU: IGF2BP3-OE enhances proliferation and reverses palbociclib inhibition (scale bar = 50 μm). (**E**) Western blot: IGF2BP3-KD downregulates MYC and targets, rescued by MYC overexpression. (**F**) Cell cycle: IGF2BP3-KD arrests cycle, rescued by MYC. (**G**,**H**) Colony formation and EdU: IGF2BP3-KD reduces proliferation, rescued by MYC (scale bar = 50 μm). The original WB membranes are provided in Supplementary WB gels. *n* = 3; mean ± SD; * *p* < 0.05, ** *p* < 0.01, *** *p* < 0.001, **** *p* < 0.0001.

To test whether IGF2BP3 is functionally required for bladder cancer proliferation and drug response, we established stable IGF2BP3-overexpressing (OE) RT112 and UMUC-3 cells using lentiviral transduction. IGF2BP3-OE robustly enhanced cell proliferation, accelerated cell-cycle progression, and increased clonogenic capacity (Figure 2A–D). Consistent with the central role of MYC in proliferative transcriptional programs, IGF2BP3-OE increased MYC abundance and elevated expression of canonical MYC downstream cell-cycle regulators, including Skp2, Cyclin E1, Cyclin A, and E2F1 (Figure 2A).

Palbociclib treatment of control cells induced a pronounced G1/S blockade, accompanied by decreased MYC and reduced expression of Skp2, Cyclin E1, Cyclin A, phosphorylated RB, and E2F1 (Figure 2A), consistent with effective CDK4/6 pathway suppression. Strikingly, forced IGF2BP3 expression partially mitigated palbociclib-induced growth inhibition and cell-cycle arrest (Figure 2A–D), indicating that IGF2BP3 can buffer the cytostatic response to CDK4/6 inhibition.

Conversely, IGF2BP3 knockdown using two independent shRNAs in RT112 and UMUC-3 markedly reduced proliferation, delayed cell-cycle progression, suppressed colony formation, and decreased MYC and downstream target expression (Figure 2E–H). To determine whether MYC is a functional effector of IGF2BP3, we reconstituted MYC in IGF2BP3-silenced cells. MYC restoration substantially rescued the proliferative defects induced by IGF2BP3-KD (Figure 2E–H). Together, these data establish IGF2BP3 as an MYC-dependent driver of proliferation and an attenuator of palbociclib-induced cytostasis in bladder cancer cells.

### 3.3. The IGF2BP3–MYC Axis Controls Tumor Growth In Vivo

**Figure 3:**
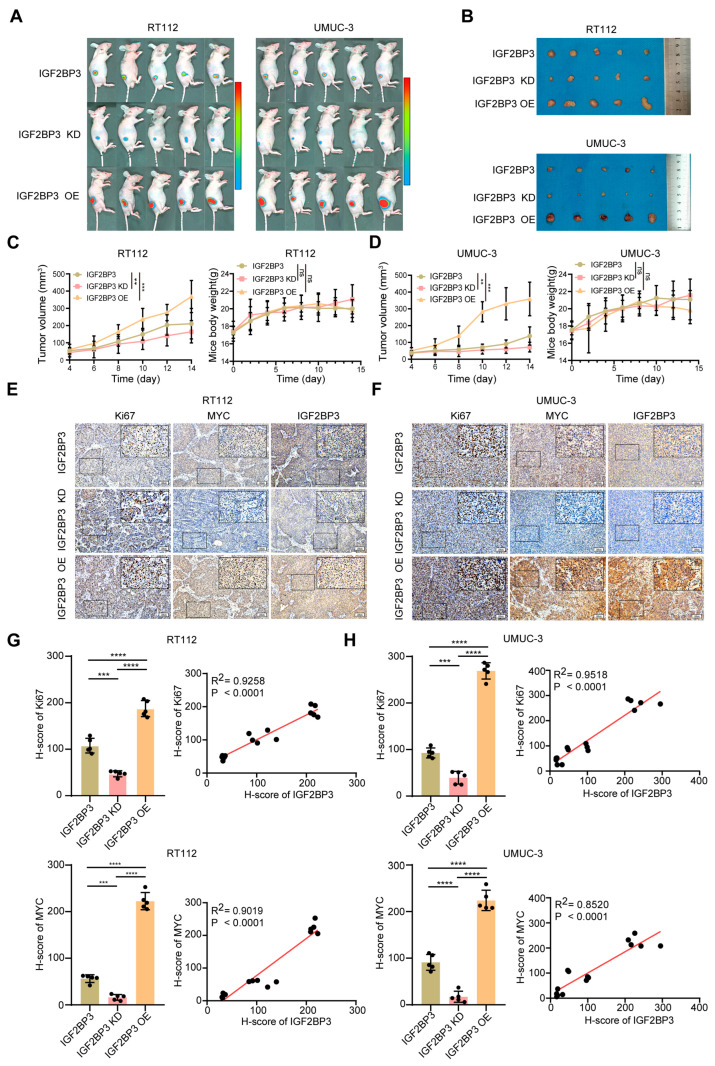
The IGF2BP3–MYC axis controls tumor growth in vivo. (**A**) Xenograft photos: tumor size varies by IGF2BP3 status (control, KD, OE). (**B**) Dissected RT112/UMUC-3 xenografts at 14 days. (**C**,**D**) Body weight gain decreases with higher IGF2BP3; IGF2BP3-KD inhibits and OE promotes tumor growth. (**E**,**F**) IHC staining shows the expression levels of IGF2BP3, MYC and Ki67 across overexpression (OE), control and knockdown (KD) groups (scale bar = 100 μm). (**G**,**H**) IGF2BP3 H-score positively correlates with MYC/Ki67 in xenografts (all *p* < 0.0001), the red lines in panels represent linear regression fit lines to show the linear correlation between the IGF2BP3 and Ki67 or MYC in scatter plot. *n* = 5/group; mean ± SD; ns, not significant; ** *p* < 0.01; *** *p* < 0.001, **** *p* < 0.0001.

We next examined whether IGF2BP3 regulates tumor growth in vivo. RT112 and UMUC-3 cells with IGF2BP3-KD, control, or IGF2BP3-OE were injected subcutaneously into nude mice to establish xenografts. All mice developed tumors at injection sites (Figure 3A). Across both models, tumor growth increased in a stepwise manner with rising IGF2BP3 expression (IGF2BP3-KD < control < IGF2BP3-OE), whereas body weight gain showed the opposite trend (Figure 3A–D). Endpoint tumor measurements confirmed that IGF2BP3 depletion significantly reduced tumor volume, while IGF2BP3-OE markedly increased tumor burden (Figure 3A–D).

Immunohistochemical analysis of xenograft tissues revealed concordant modulation of IGF2BP3, MYC, and Ki67: expression of all three markers decreased in IGF2BP3-KD tumors and increased in IGF2BP3-OE tumors (Figure 3E,F). Quantification further showed that Ki67 and MYC levels positively correlated with IGF2BP3 abundance across tumors (Figure 3G,H). These in vivo data validate the functional importance of the IGF2BP3–MYC axis in sustaining bladder tumor proliferation.

### 3.4. IGF2BP3 Facilitates MYC Expression in Bladder Cancer Cells Through Recognition of m^6^A Modification

**Figure 4:**
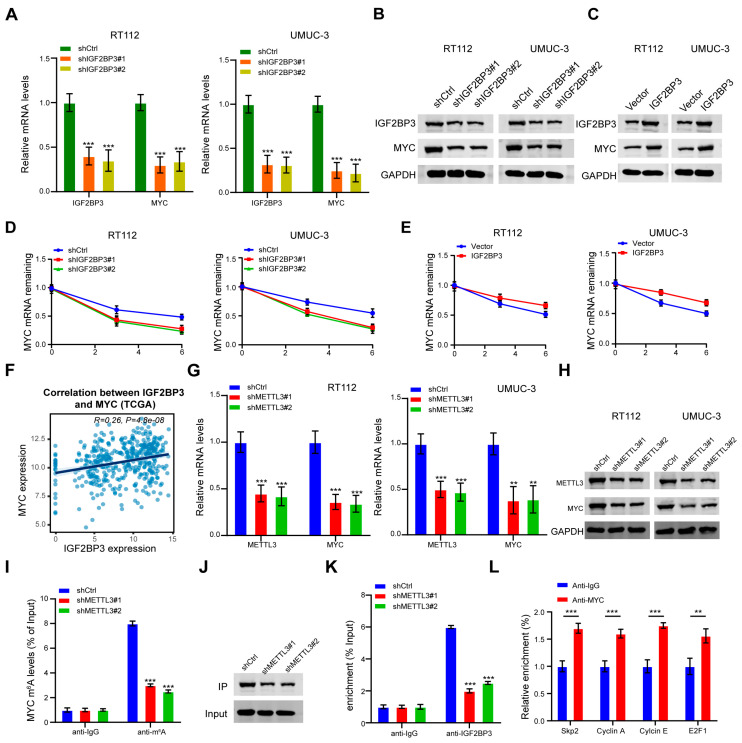
IGF2BP3 facilitates MYC expression in bladder cancer cells through recognition of m^6^A modification. (**A**,**B**) IGF2BP3-KD reduces *MYC* mRNA/protein. (**C**) IGF2BP3 modulation alters MYC protein levels. (**D**,**E**) IGF2BP3 stabilizes *MYC* mRNA. (**F**) TCGA: *IGF2BP3* positively correlates with *MYC*. (**G**,**H**) METTL3-KD reduces MYC expression. (**I**,**J**) Me-RIP/IP: METTL3-KD lowers *MYC* mRNA m^6^A level. (**K**) RIP: METTL3-KD reduces IGF2BP3-MYC mRNA binding. (**L**) ChIP-qPCR: MYC binds target gene promoters. The original WB membranes are provided in Supplementary WB gels. Mean ± SD; ** *p* < 0.01, *** *p* < 0.001.

Previous studies have shown that IGF2BP3 acts as an m^6^A reader to enhance *MYC* expression by stabilizing *MYC* mRNA [18,23]; however, whether this mechanism operates in bladder cancer (BLCA) remains unclear. To address this, we investigated the role of IGF2BP3 in MYC regulation in BLCA. qRT–PCR and Western blot analyses revealed that IGF2BP3 knockdown significantly decreased *MYC* mRNA and protein levels, whereas its overexpression increased MYC expression (Figure 4A–C), indicating a positive regulatory effect. Consistent with the role of IGF2BP proteins in stabilizing m^6^A-modified transcripts [17,24], Actinomycin D assays showed that MYC mRNA decay was accelerated upon IGF2BP3 depletion but prolonged following its overexpression (Figure 4D,E). TCGA analysis further demonstrated a statistically significant positive correlation between *IGF2BP3* and *MYC* expression in BLCA (Pearson’s R = 0.26, *p* = 4.8 × 10^−8^ (Figure 4F)). To determine whether this regulation is m6A-dependent, we examined METTL3, a key m^6^A methyltransferase [25]. METTL3 knockdown reduced *MYC* mRNA and protein levels (Figure 4G,H) and decreased m^6^A enrichment on *MYC* transcripts, as shown by m6A-RIP assays (Figure 4I,J). RIP assays further revealed that IGF2BP3 binding to *MYC* mRNA was diminished upon METTL3 depletion (Figure 4K), indicating m^6^A dependence. This IGF2BP3-MYC regulatory axis has been similarly documented in other malignancies, such as osteosarcoma, where IGF2BP3 stabilizes *MYC* mRNA in an m6A-dependent manner [26], given that MYC is a central regulator of cell-cycle progression [27,28]. To further confirm that the IGF2BP3-mediated increase in MYC protein translates into enhanced transcriptional activity, ChIP–qPCR confirmed MYC binding to the promoters of its canonical target genes *Skp2*, *Cyclin A*, *Cyclin E* and *E2F1* (Figure 4L). Collectively, these results suggest that IGF2BP3 promotes cell-cycle progression and proliferation in BLCA by stabilizing m^6^A-modified *MYC* transcripts.

### 3.5. MKRN2 Mediates Ubiquitination and Proteasomal Degradation of IGF2BP3 in Bladder Cancer Cells

**Figure 5:**
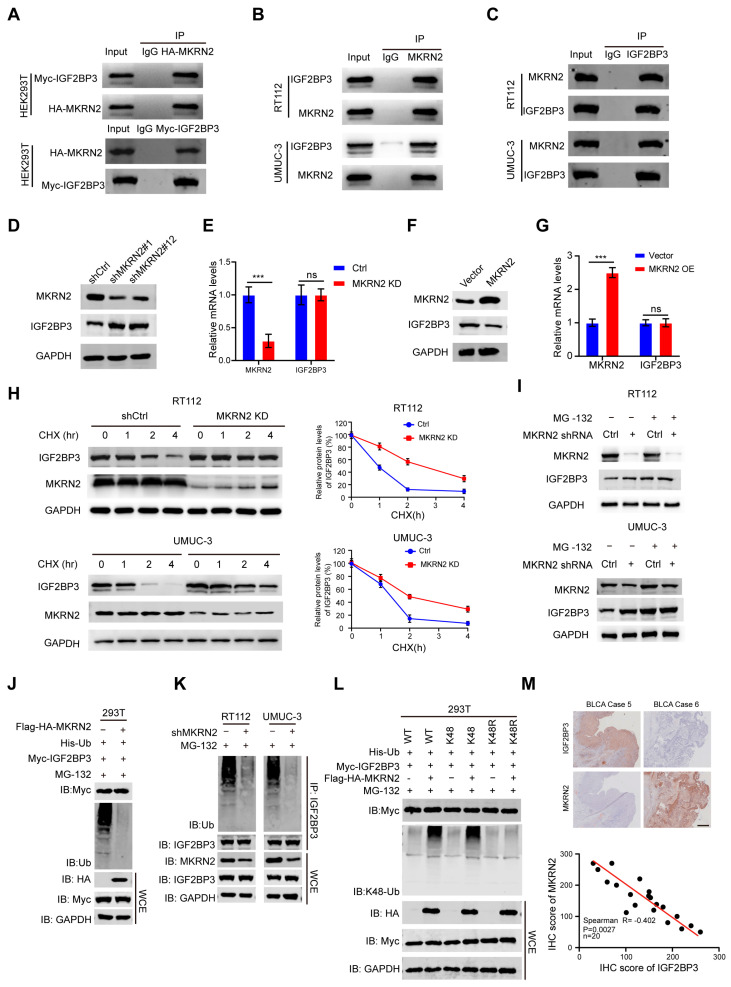
MKRN2 mediates ubiquitination and proteasomal degradation of IGF2BP3 in bladder cancer cells. (**A**) Exogenous Co-IP: Myc-IGF2BP3 interacts with HA-MKRN2. (**B**,**C**) Endogenous Co-IP: IGF2BP3 binds MKRN2 in RT112/UMUC-3. (**D**,**E**) MKRN2-KD increases IGF2BP3 protein (mRNA unchanged). (**F**,**G**) MKRN2-OE reduces IGF2BP3 protein. (**H**) CHX chase: MKRN2-KD prolongs IGF2BP3 half-life. (**I**) MG-132 blocks MKRN2-mediated IGF2BP3 degradation. (**J**) In vivo ubiquitination: MKRN2 promotes IGF2BP3 ubiquitination. (**K**) MKRN2-KD increases IGF2BP3 ubiquitination. (**L**) K48-linked ubiquitination required for IGF2BP3 degradation. (**M**) Clinical samples: IGF2BP3 inversely correlates with MKRN2, the red lines in panels represent linear regression fit lines to show the linear correlation between the IGF2BP3 and MKRN2 in scatter plot. (R = −0.402, *p* = 0.0027; scale bar = 200 μm). The original WB membranes are provided in Supplementary WB gels. Mean ± SD; ns, not significant; *** *p* < 0.001.

Although IGF2BP3 stabilizes MYC in an m^6^A-dependent manner and promotes proliferation in BLCA, the mechanism underlying its protein upregulation remains unclear. As ubiquitin-mediated proteasomal degradation is a key pathway regulating protein homeostasis, we screened potential E3 ubiquitin ligases and identified MKRN2 as a candidate mediating IGF2BP3 degradation [20,21]. Co-immunoprecipitation assays confirmed physical binding between IGF2BP3 and MKRN2 (Figure 5A–C). Functionally, MKRN2 knockdown significantly increased IGF2BP3 protein levels without altering its mRNA expression (Figure 5D,E), suggesting post-translational regulation. Conversely, MKRN2 overexpression resulted in decreased IGF2BP3 protein levels, while IGF2BP3 mRNA remained unchanged (Figure 5F,G), supporting a proteostatic mechanism. CHX chase assays revealed that IGF2BP3 protein turnover was markedly slowed upon MKRN2 depletion (Figure 5H), and proteasome inhibition by MG-132 abrogated MKRN2-mediated IGF2BP3 degradation (Figure 5I), indicating that MKRN2 regulates IGF2BP3 stability via the ubiquitin–proteasome system. This is consistent with established roles of E3 ligases in substrate degradation [29,30]. Ubiquitination assays further confirmed that MKRN2 promotes polyubiquitination of IGF2BP3 (Figure 5J), verifying MKRN2 as a bona fide E3 ligase for IGF2BP3. In BLCA cells, MKRN2 knockdown similarly increased IGF2BP3 protein levels (Figure 5K), demonstrating that this regulatory axis operates in tumor contexts. MKRN2 facilitated the attachment of K48-linked polyubiquitin chains to IGF2BP3, which target substrates for proteasomal degradation. By contrast, substitution of wild-type ubiquitin with the K48R mutant (K48 mutated to R) blocked MKRN2-induced ubiquitination of IGF2BP3 in 293T cells. These data indicate that MKRN2 interacts with IGF2BP3 and promotes its K48-linked polyubiquitination, leading to its proteasomal degradation (Figure 5L). Consistent with these biochemical findings, functional rescue experiments demonstrated that MKRN2 overexpression inhibited IGF2BP3-induced MYC expression and downstream cell-cycle regulators including Skp2, Cyclin A, Cyclin E, and E2F1 (Supplementary Figure S2A). Flow cytometry revealed that MKRN2 overexpression reversed the increase in S-phase cell population induced by IGF2BP3 overexpression (Supplementary Figure S2B), while colony formation and EdU incorporation assays further confirmed that MKRN2 attenuated IGF2BP3-mediated proliferative effects (Supplementary Figure S2C,D). Importantly, immunohistochemical analysis of BLCA clinical specimens revealed a significant inverse correlation between IGF2BP3 and MKRN2 expression (Spearman R = −0.402, *p* = 0.0027, *n* = 20; Figure 5M), underscoring the physiological relevance of this regulatory mechanism in vivo. Together, these data indicate that MKRN2 modulates IGF2BP3 protein stability through ubiquitination and proteasomal degradation, acting as a negative regulator of IGF2BP3 in bladder cancer.

### 3.6. MKRN2 Suppresses MYC Expression by Degrading IGF2BP3 and Synergizes with Palbociclib to Inhibit Bladder Cancer Progression In Vivo

**Figure 6:**
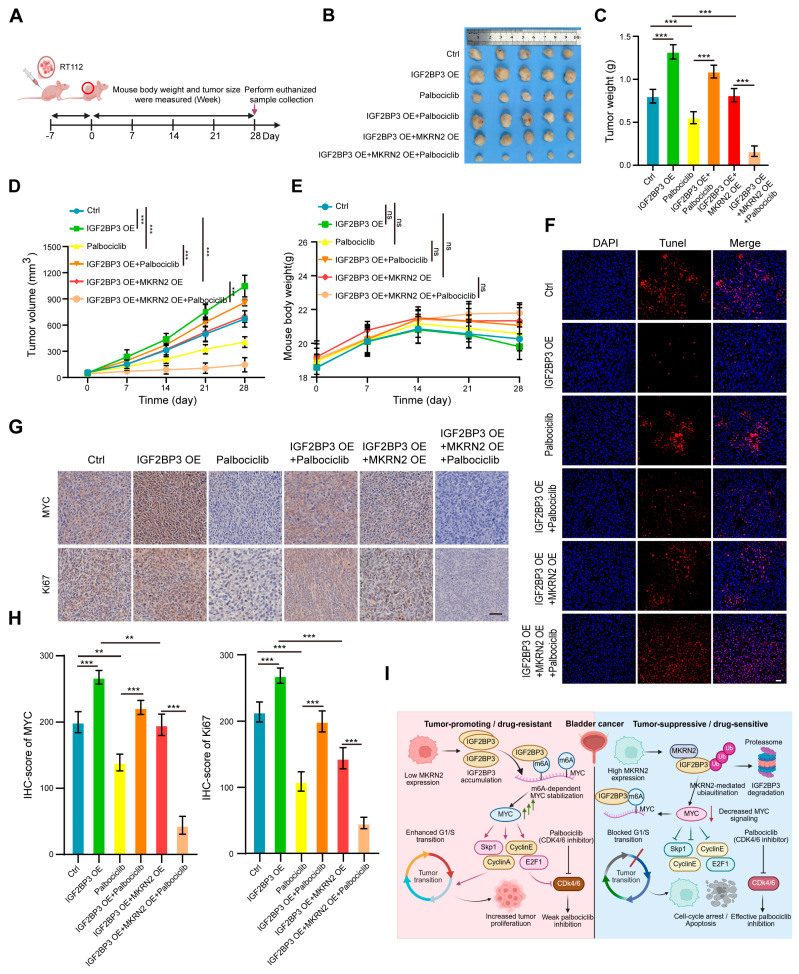
MKRN2 suppresses MYC expression by degrading IGF2BP3 and synergizes with palbociclib to inhibit bladder cancer progression in vivo. (**A**) Xenograft experimental design, the red circles represent tumor cells. (**B**) Representative tumor photos per group. (**C**) Tumor weight statistics. (**D**) Tumor volume growth curves. (**E**) Mouse body weight curves. (**F**) TUNEL staining: apoptosis detection (red = TUNEL+, blue = DAPI; scale bar = 50 μm). (**G**) IHC: MYC expression in tumors (scale bar = 50 μm). (**H**) IHC: Ki67 expression in tumors. (**I**) Mechanistic model: MKRN2 ubiquitinates/degrades IGF2BP3, suppressing MYC; MKRN2-OE synergizes with palbociclib. Mean ± SD; ns, not significant; ** *p* < 0.01, *** *p* < 0.001.

To investigate whether MKRN2 suppresses MYC axis activity by degrading IGF2BP3 and enhances the in vivo efficacy of CDK4/6 inhibition, we established RT112 cell-derived xenograft models with six groups: control, IGF2BP3 overexpression (OE), palbociclib alone, IGF2BP3 OE combined with palbociclib, IGF2BP3 OE combined with MKRN2 OE, and the triple combination group (IGF2BP3 OE + MKRN2 OE + palbociclib) (Figure 6A). Compared with the control group, the IGF2BP3 OE group exhibited significantly increased tumor volume and weight, whereas palbociclib monotherapy only partially suppressed tumor growth. Compared with the IGF2BP3 OE group, both the IGF2BP3 OE + palbociclib and IGF2BP3 OE + MKRN2 OE groups showed reduced tumor growth, indicating that MKRN2 OE effectively antagonizes IGF2BP3-driven tumor progression. Notably, compared with either the IGF2BP3 OE + palbociclib group or the IGF2BP3 OE + MKRN2 OE group, the triple combination group (IGF2BP3 OE + MKRN2 OE + palbociclib) exhibited the most pronounced suppression of tumor volume and weight (Figure 6B–D). No significant differences in body weight were observed among the groups, indicating favorable safety profiles (Figure 6E). Tunel staining revealed that, compared with the control group, the IGF2BP3 OE group had fewer apoptotic cells; compared with the IGF2BP3 OE group, all treatment groups showed increased apoptosis, with the triple combination group exhibiting the highest level of apoptosis (Figure 6F). Immunohistochemical analysis showed that, compared with the control group, MYC and Ki67 expression was upregulated in the IGF2BP3 OE group; compared with the IGF2BP3 OE group, the IGF2BP3 OE + palbociclib, IGF2BP3 OE + MKRN2 OE, and triple combination groups all showed reduced MYC and Ki67 expression, with the most significant reduction observed in the triple combination group (Figure 6G,H). Collectively, these findings demonstrate that MKRN2 downregulates MYC expression by promoting IGF2BP3 degradation and synergizes with palbociclib to suppress bladder cancer progression in vivo (Figure 6I).

## 4. Discussion

This study defines the MKRN2–IGF2BP3–MYC axis as a key regulator of proliferation and CDK4/6 inhibitor sensitivity in bladder cancer, providing new insights into m^6^A reader networks and therapeutic resistance.

IGF2BP3, a canonical m^6^A reader, is upregulated in multiple cancers and stabilizes oncogenic mRNAs [17,18]. Here, *IGF2BP3* was elevated in bladder cancer, correlated with poor prognosis and Ki67 positivity, and was the only m^6^A regulator induced by palbociclib. Functional assays confirmed that IGF2BP3 overexpression rescued palbociclib-induced G1/S arrest, while IGF2BP3 knockdown suppressed proliferation in an MYC-dependent manner.

Mechanistically, our study demonstrates that *IGF2BP3* promotes bladder cancer progression and CDK4/6 inhibitor resistance by enhancing *MYC* mRNA stability through m^6^A-dependent recognition, thereby sustaining MYC signaling. This finding is consistent with previous studies showing that IGF2BP family proteins stabilize m^6^A-modified transcripts to promote oncogenic gene expression [17,24], including regulation of FZD1/7 in triple-negative breast cancer [31]. Given the broad role of m^6^A modifications in bladder cancer progression [14,32,33], our results further identify MYC as an important downstream effector linking m^6^A-dependent RNA regulation to cell-cycle activation and therapeutic resistance. More importantly, we uncovered a previously unrecognized layer of IGF2BP3 regulation by identifying MKRN2 as a negative regulator of IGF2BP3 protein stability through ubiquitin-mediated proteasomal degradation. While post-translational regulation of IGF2BP3 has recently been reported through USP10-mediated deubiquitination in non-small cell lung cancer [34], our findings establish MKRN2 as an E3 ubiquitin ligase that antagonizes IGF2BP3 accumulation. Together with previous observations in neuroblastoma [21], these results support the existence of a conserved MKRN2–IGF2BP3 regulatory axis while extending its functional significance to bladder cancer and CDK4/6 inhibitor resistance.

From a therapeutic perspective, the MKRN2–IGF2BP3–MYC axis represents a potential vulnerability for overcoming resistance to CDK4/6 inhibitors. Restoring MKRN2 activity or promoting IGF2BP3 degradation may suppress persistent MYC signaling and enhance the efficacy of CDK4/6 inhibition. This concept is supported by our in vivo findings and is complementary to previously reported mechanisms of CDK4/6 inhibitor resistance, including compensatory activation of PI3K/AKT/mTOR and RTK signaling pathways [35,36], as well as MYC-mediated RB1 degradation [14]. Therefore, our study expands the molecular network underlying CDK4/6 inhibitor resistance and provides a rationale for therapeutic strategies targeting the MKRN2–IGF2BP3–MYC signaling axis in bladder cancer.

Beyond targeted therapy, our findings may also have implications for cancer immunotherapy. IGF2BP3 has been recognized as an oncofetal antigen with restricted expression in normal adult tissues and aberrant overexpression in multiple malignancies, making it a promising candidate for therapeutic cancer vaccine development [37]. Recent advances in cancer vaccine platforms, particularly mRNA- and peptide-based vaccines, have highlighted the potential of combining tumor-specific antigens with molecularly targeted therapies to improve anti-tumor immune responses [38]. Therefore, modulation of the MKRN2–IGF2BP3–MYC axis may not only suppress tumor proliferation but also enhance the efficacy of cancer vaccines or other immunotherapeutic strategies. Although this possibility remains speculative and requires further experimental validation, our study provides a mechanistic rationale for exploring IGF2BP3-targeted vaccines in combination with CDK4/6 inhibitors in bladder cancer.

Several limitations should be acknowledged. First, the upstream regulation of MKRN2 remains unclear, and additional IGF2BP3 targets may also contribute to drug resistance. Second, our findings from subcutaneous xenograft models require validation in orthotopic or organoid models. Third, the survival analysis was based on TCGA overall survival data rather than bladder cancer-specific survival, and the lack of comprehensive comorbidity information in the TCGA dataset may have influenced the prognostic analysis. Therefore, the prognostic value of IGF2BP3 warrants further validation in prospective cohorts with more comprehensive clinical data. Finally, the correlation analysis between MKRN2 and IGF2BP3 was performed in a relatively small clinical cohort (*n* = 20). Although a significant inverse correlation was observed, larger multicenter cohorts are required to further validate the robustness and clinical significance of this finding.

## 5. Conclusions

In conclusion, this study establishes the MKRN2–IGF2BP3–MYC axis as a critical regulator of CDK4/6 inhibitor response in bladder cancer and reveals a novel mechanism by which MKRN2 suppresses MYC expression and enhances therapeutic sensitivity through degradation of IGF2BP3. These findings provide a rationale for molecular stratification, response prediction, and combination targeted therapy in bladder cancer.

## Data Availability

De-identified clinicopathologic data may be available from the corresponding author upon reasonable request and in accordance with institutional and ethical regulations.

## Supplementary Materials

The following supporting information can be downloaded at: https://www.mdpi.com/article/10.3390/cancers18132164/s1, Figure S1: Transcriptomic profiling identified IGF2BP3 as a CDK4/6 inhibitor-responsive m^6^A regulatory gene. (A) Experimental design: 3 cell lines, 6 groups (control/1 μM palbociclib, 48 h), RNA-seq. (B) Gene expression distribution across samples. (C) DEG heatmaps (top 25 up/down) for paired comparisons; Figure S2: MKRN2 overexpression inhibits IGF2BP3-induced MYC expression and cell-cycle progression. (A) Western blot: MKRN2-OE reverses IGF2BP3-induced MYC/target upregulation. (B) Cell cycle: MKRN2-OE reverses IGF2BP3-induced S-phase increase. (C,D) Colony formation/EdU: MKRN2-OE attenuates IGF2BP3-driven proliferation. Mean ± SD; ** *p* < 0.01, *** *p* < 0.001. Table S1: Clinical sample information of bladder cancer patients; Table S2: An overview of m^6^A modification regulatory molecules: writer, eraser, and reader families.

## Abbreviations

The following abbreviations are used in this manuscript:

BLCA	Bladder cancer
CDK4/6	Cyclin-dependent kinase 4/6
ChIP	Chromatin immunoprecipitation
CHX	Cycloheximide
Co-IP	Co-immunoprecipitation
DEGs	Differentially expressed genes
EdU	5-Ethynyl-2′-deoxyuridine
FBS	Fetal bovine serum
HRP	Horseradish peroxidase
IHC	Immunohistochemistry
KD	Knockdown
m^6^A	N^6^-methyladenosine
Me-RIP	Methylated RNA immunoprecipitation
OE	Overexpression
OS	Overall survival
PBS	Phosphate-buffered saline
PVDF	Polyvinylidene fluoride
qRT-PCR	Quantitative real-time polymerase chain reaction
RIPA	Radioimmunoprecipitation assay
RIP	RNA immunoprecipitation
SDS-PAGE	Sodium dodecyl sulfate–polyacrylamide gel electrophoresis
TCGA	The Cancer Genome Atlas
TUNEL	Terminal deoxynucleotidyl transferase dUTP nick end labeling
WB	Western blot
