# Supplementary material for: MKRN2-Mediated Degradation of IGF2BP3 Suppresses MYC and Enhances CDK4/6 Inhibitor Sensitivity in Bladder Cancer
Source: Cancers (Basel). 2026 Jul 6;18(13):2164. doi: 10.3390/cancers18132164 (PMC13359444; doi:10.3390/cancers18132164)
Supplement: Supplementary file 1 [file cancers-18-02164-s001.zip › Supplementary table S1.pdf]

Cases	Age (years)	Gender	TNM stage	Paracancerous tissue	Neoadjuvant
Patient 1	55	Male	T2aN0M0	Yes	GC
Patient 2	65	Female	T2bN0M0	Yes	None
Patient 3	78	Male	T2aN0M0	Yes	None
Patient 4	82	Male	T2bN0M0	Yes	None
Patient 5	62	Male	T2bN0M0	Yes	None
Patient 6	74	Male	T2bN1M0	Yes	None
Patient 7	72	Female	T2bN0M0	Yes	GC
Patient 8	56	Female	T2bN0M0	Yes	None
Patient 9	59	Male	T2aN0M0	Yes	GC
Patient 10	63	Male	T2bN0M0	Yes	GC
Patient 11	81	Male	T2aN0M0	Yes	None
Patient 12	74	Male	T2aN0M0	Yes	None
Patient 13	71	Female	T2aN0M0	Yes	None
Patient 14	68	Male	T3N1M0	Yes	GC
Patient 15	68	Female	T2aN0M0	Yes	None
Patient 16	72	Male	T2aN0M0	Yes	None
Patient 17	72	Male	T2bN0M0	Yes	None
Patient 18	76	Male	T3N1M0	Yes	GC
Patient 19	69	Female	T3N1M0	Yes	GC
Patient 20	53	Male	T2bN0M0	Yes	None
**Abbreviations: M, male; F, female; GC, Gemcitabine Cisplatin.**					
