# Supplementary material for: MKRN2-Mediated Degradation of IGF2BP3 Suppresses MYC and Enhances CDK4/6 Inhibitor Sensitivity in Bladder Cancer
Source: Cancers (Basel). 2026 Jul 6;18(13):2164. doi: 10.3390/cancers18132164 (PMC13359444; doi:10.3390/cancers18132164)
Supplement: Supplementary file 1 [file cancers-18-02164-s001.zip › Supplementary table S2.pdf]

	Name
**Writers**	METTL3, METTL14, METTL16, METTL5, ZCCHC4, WTAP, VIRMA, ZC3H14, RBM15, RBM15B
**Erasers**	ALKBH5, FTO
**Readers**	YTHDF1, YTHDF2, YTHDF3, YTHDC1, YTHDC2, IGF2BP1, IGF2BP3, IGF2BP3, eIF3b, eIF3h, hnRNPA2B1, hnRNPC
