# Supplementary figures and images for: MKRN2-Mediated Degradation of IGF2BP3 Suppresses MYC and Enhances CDK4/6 Inhibitor Sensitivity in Bladder Cancer
Source: Cancers (Basel). 2026 Jul 6;18(13):2164. doi: 10.3390/cancers18132164 (PMC13359444; doi:10.3390/cancers18132164)

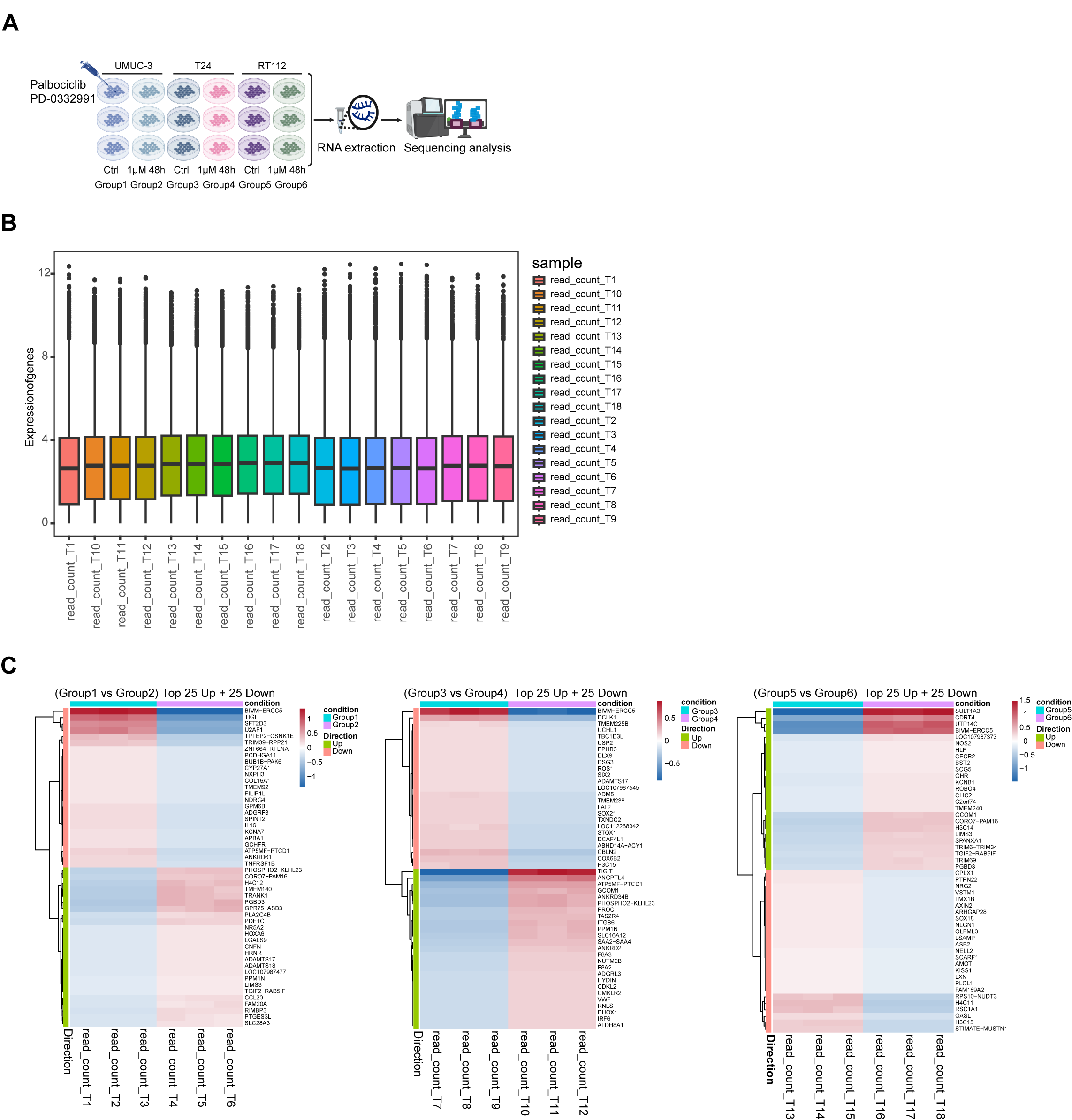

Supplement: Supplementary file 1 [file cancers-18-02164-s001.zip › Supplementary figure S1.tif]

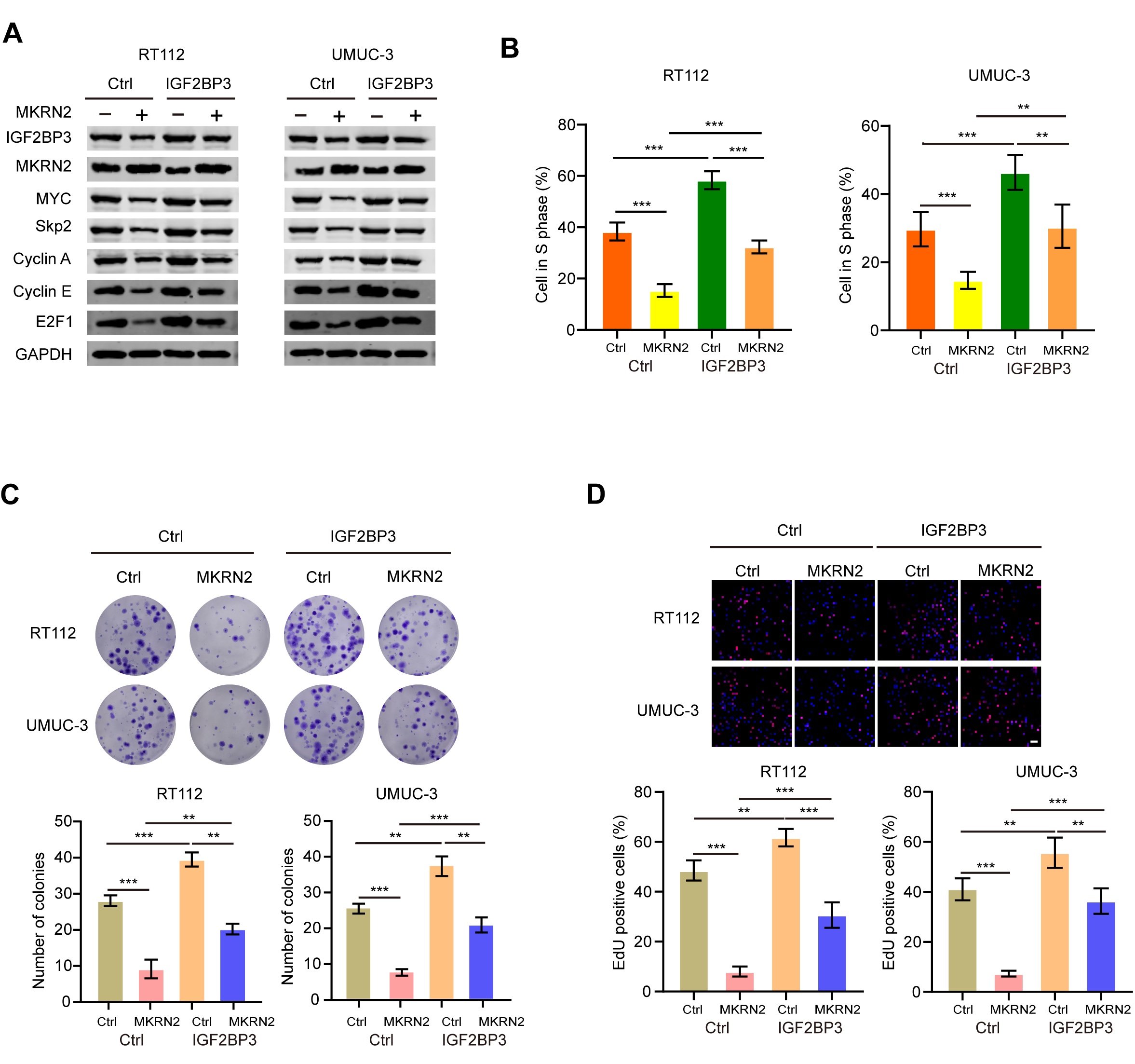

Supplement: Supplementary file 1 [file cancers-18-02164-s001.zip › Supplementary figure S2.tif]
